# Cell-Laden Gel Biomimetic Skin Promotes Full-Thickness Skin Wound Regeneration

**DOI:** 10.3390/gels12030258

**Published:** 2026-03-20

**Authors:** Pei Zhang, Qianqian Chen, Yuge Pu, Mingxing Liu, Mengru Ma, Yihan Wu, Ying Zhang, Xueyi Yang

**Affiliations:** College of Life Sciences, Luoyang Normal University, Luoyang 471934, China

**Keywords:** biomimetic skin, EpiSCs, amniotic membrane matrix, collagen gel, hierarchical regeneration

## Abstract

The regeneration and repair of scarless skin tissue remain a significant challenge for full-thickness wounds. Traditional wound management approaches, particularly passive healing through scabbing and conventional mechanical debridement, are frequently associated with significant pain, high infection risks, and abnormal scar formation, often failing to support the regeneration of skin appendages like hair follicles. In recent years, collagen-based scaffolds have been widely adopted in tissue-engineered skin substitutes owing to their favorable biocompatibility. However, their simplistic, single-component architecture inherently lacks the dynamic, cell-instructive microenvironment found in native skin, which not only compromises the long-term survival and functional integration of seeded cells but also directly leads to insufficient reconstruction of the dermo-epidermal junction, thereby impairing skin barrier function and ultimately limiting overall regenerative efficacy. In this study, we propose a biomimetic multilayer composite scaffold system in which decellularized amniotic membrane matrix (AM) is combined with fibroblast-laden collagen gel (FCG) and seeded with epidermal stem cells (EpiSCs). This bionic skin (denoted as AM-FCG-EpiSCs) is designed to achieve hierarchical regeneration of full-thickness skin defects. Compared with injured skin treated with Moropicin ointment, the injured skin treated with AM-FCG-EpiSCs healed more quickly and regenerated appendages like hair follicles without scarring. The results show that the biomimetic structure of AM-FCG-EpiSCs can mediate dynamic cell–cell interactions and regulate the microenvironment. This breakthrough overcomes the dual challenges of scar suppression and functional restoration in full-thickness skin regeneration, offering an innovative solution for translational medicine.

## 1. Introduction

The skin, the largest organ in both animals and humans, acts as the primary barrier against external microbial invasion and various physical and chemical injuries [1]. Damage to the skin can lead to a decline in immune function and an increased risk of infection. Severe skin damage may also cause long-term pain, fibrosis, and difficult-to-heal ulcers, significantly affecting the quality of life of patients [2]. Recent years have witnessed remarkable advancements in the treatment of skin injuries through the introduction of artificial skin into clinical practice. Nevertheless, numerous challenges remain to be addressed, and we are still far from achieving the ideal goal of “perfect restoration”. The mechanical properties of natural biomaterials are inadequate to withstand external forces, rendering them susceptible to deformation or damage. Moreover, their rapid degradation prevents them from lasting long enough in the body, undermining the therapeutic efficacy [3]. On the other hand, synthetic biomaterials exhibit limited biocompatibility, potentially triggering immune rejection. Additionally, their slow degradation rate can readily result in side effects [4].

It is well-established that stem cell therapy has shown significant potential in wound healing and is regarded as one of the important directions for future wound treatment in clinical practice. However, simple stem cell therapy has some drawbacks, such as the potential for cell damage, loss and limited therapeutic efficacy. Excessive activation of dermal fibroblasts (FBs) results in disordered extracellular matrix (ECM) secretion, leading to pathological scar formation, while skin appendages like hair follicles are permanently lost due to the absence of a regenerative microenvironment [5]. Research has confirmed that the microenvironment plays a crucial role in maintaining stem cell potency and guiding directed differentiation [6]. Existing artificial skin still exhibits significant shortcomings, particularly in the inadequate integration of its mechanical support structure and bioactive components. It fails to effectively mimic dermal tissue or reconstruct a regenerative microenvironment conducive to intercellular signaling networks. This limitation hinders stem cell proliferation and differentiation, severely impacting the efficacy of wound repair and functional reconstruction. In skin tissue engineering, the amniotic membrane (AM) is widely used as a scaffold material due to its ability to mimic the stem cell niche. For instance, Hashemi et al. transplanted acellular amniotic membranes loaded with dermal FBs and umbilical cord mesenchymal stem cells into chronic wounds of diabetic patients, achieving significant wound healing effects [7]. However, AM inherently lacks sufficient thickness and mechanical strength to fully meet the structural requirements for dermal layer reconstruction [8]. To address this issue, our group proposed a composite strategy: using AM as the bioactive basal layer and introducing a dense three-dimensional network gel layer formed by collagen molecular self-assembly on top of it [9].

Pure collagen, on the other hand, is mechanically weak and prone to rupture under minimal tension. In response to this, Ding introduced high-density FBs into the collagen, and this gel composite material demonstrated excellent performance in stimulating collagen regeneration and constructing supportive structures [10]. Through this approach, the inert collagen gel (CG) was transformed into a bioactive, fibroblast-laden collagen gel (FCG). Delayed or impaired epidermal regeneration leads to the absence of anti-fibrotic signals like TGF-β3 and IL-10. The persistent inflammatory microenvironment and abnormal mechanical tension jointly drive FBs into a pro-scarring phenotype. Due to their self-renewal and multi-lineage differentiation capabilities, as well as their ability to secrete bioactive factors like growth factors, cytokines, and exosomes, stem cells have become highly promising candidates for skin regeneration [11,12]. With this goal in mind, we also incorporated EpiSCs. These cells reside in the basal layer of the epidermis and play a significant role in hair follicle development and regeneration. They are capable of differentiating into multiple cell types within the hair follicle and secreting cytokines and signaling molecules [13].

The bioactive components within the AM can modulate Wnt/β-catenin pathway activity, thereby influencing stem cell fate [14]. When EpiSCs are seeded onto the AM surface, a subset of cells is expected to differentiate upward into the epidermis, promoting rapid wound closure. Another subset may migrate downward into the FCG, where they interact with FBs through multiple signaling pathways, establishing a bidirectional regulatory relationship [15]. For instance, during tissue injury, KGF secreted by FBs can activate epidermal keratinocytes, prompting them to release TGF-β1. Keratinocyte-derived TGF-β1 then activates downstream SMAD signaling in FBs in a paracrine manner, inducing their differentiation into myofibroblasts. These cells subsequently synthesize type I collagen and promote wound contraction, forming a positive feedback loop that collectively mediates the wound healing process [16].

**Figure 1:**
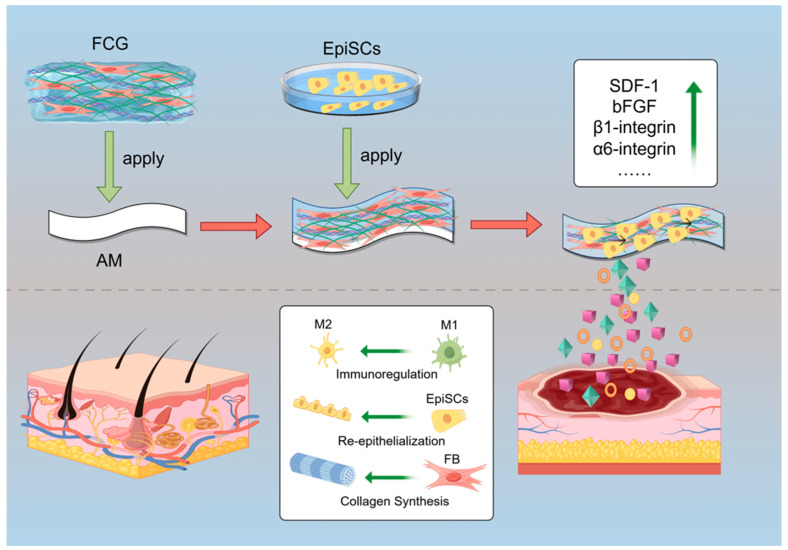
Schematic diagram of the fabrication process of the AM-FCG-EpiSCs. AM: Amniotic membrane; FCG: Fibroblast-laden collagen gel; EpiSCs: Epidermal stem cells (Created with Figdraw).

Herein, we propose a novel artificial skin construction strategy: using AM as a scaffold, FCG forms the dermal layer and integrates with the epidermal layer formed by EpiSCs to construct a bionic composite skin system (Figure 1). Within this system, FBs synthesize ECM to provide a scaffold for the new blood vessels and EpiSCs migration, playing a crucial role in dermal layer repair. EpiSCs differentiate into epidermal cells and secrete cytokines, replenishing the damaged epidermis layer while inhibiting FBs from adopting a pro-scarring phenotype. Through bidirectional regulation, these two cell types collaboratively promote the healing of full-thickness skin wounds and improve repair outcomes, with the ultimate goal of achieving scarless repair and hair follicle regeneration in full-thickness skin defects. Therefore, we posit that the underlying principle of this biomimetic skin holds significant potential to advance the fields of skin regeneration and tissue engineering.

## 2. Results and Discussion

### 2.1. Proliferation, Purification and Characterization of EpiSCs

**Figure 2:**
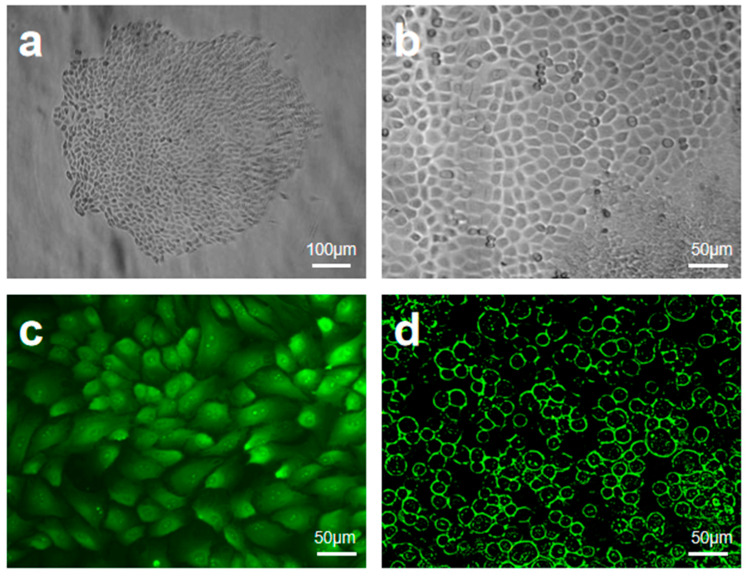
Proliferation, purification and characterization of EpiSCs: (**a**) primary epithelial cell clones; (**b**) purified epidermal stem cells; (**c**) *P63* staining; (**d**) β1-integrin positivity.

EpiSCs were successfully isolated and purified from primary epithelial cells by differential adhesion combined with selective culture medium. In primary culture, typical epithelial cell clones were observed (Figure 2a), with their edges exhibiting a “cobblestone” appearance, suggesting active cell–matrix interactions and significant proliferative potential. After purification (Figure 2b), the morphology of EpiSCs tended to be uniform, with a significantly higher nucleo-cytoplasmic ratio, consistent with the characteristics of stem cell quiescence. Further phenotypic analysis revealed a high proportion of *P63*^+^ cells in the purified EpiSCs (Figure 2c), indicating their preservation of high stemness. And its high expression of β1-integrin (Figure 2d) indicates a strong extracellular matrix adhesion function, which is consistent with the ability to anchor directionally to the basement membrane. The above characteristics indicate that purified EpiSCs can not only provide a high-quality seed cell source for multilayered artificial skin, but also their β1-integrin-mediated adhesion can significantly enhance the efficiency of bio-integration with AM-FCG after transplantation, which can lay a cytological foundation for functional skin regeneration.

Unlike the cell-free strategy used in regenerative-direction artificial skin (RDAS) [17], the biomimetic multilayer composite scaffold system developed in this study employs EpiSCs to directly supply a source of regenerative cells. While both systems enable rapid, scar-free healing and regeneration of hair follicles and sebaceous glands in full-thickness wounds, there are fundamental differences in their mechanisms of action: RDAS depends on the programmability of DNA hydrogels to induce a regenerative phenotype in FBs, whereas our system drives regeneration through the directed differentiation of stem cells in synergy with the ECM. Stem cells may possess more significant physiological advantages in areas such as sebum secretion from the perspective of long-term functional maintenance. However, their specific regulatory mechanisms and sweat gland regeneration potential still require further investigation.

### 2.2. Formation and Preliminary Characterization of AM-FCG-EpiSCs

**Figure 3:**
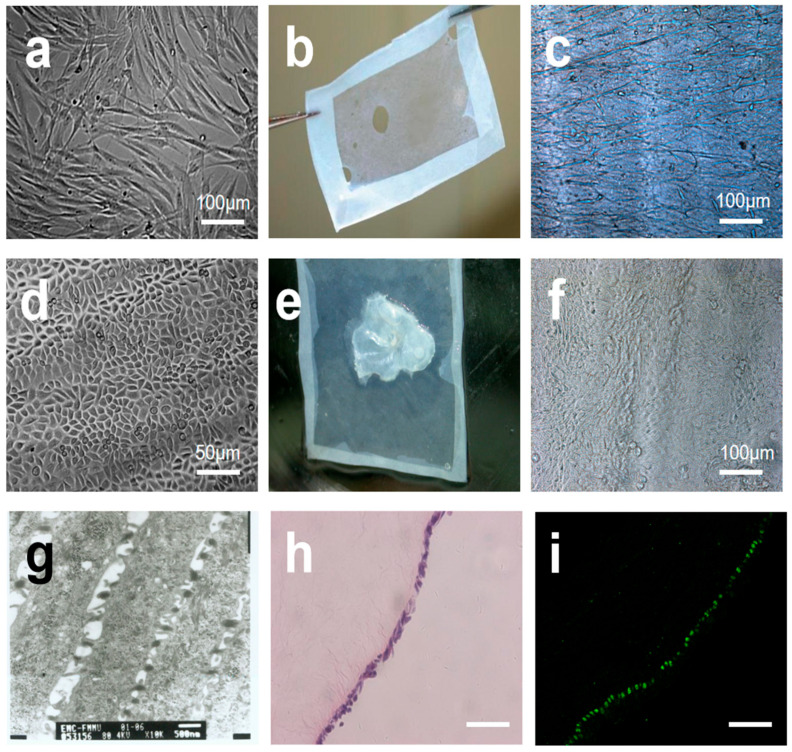
Formation and preliminary characterization of AM-FCG-EpiSCs: (**a**) fibroblasts; (**b**) decellularized amniotic membrane; (**c**) growth of collagen fibroblast gel on amniotic membrane; (**d**) epidermal stem cells; (**e**) after seeding with epidermal stem cells; (**f**) cell proliferation and differentiation following 7 days of culture; (**g**) TEM of artificial skin after 7 days of culture; (**h**) H&E staining of artificial skin after 7 days of culture; (**i**) fluorescence microscopy imaging of artificial skin based on *P63* protein expression after 7 days of culture. All scale bars represent 100 µm.

FBs exhibited typical spindle-shaped adherent growth with uniform cytoplasmic stretch (Figure 3a). FCG was inoculated on the AM (Figure 3b) surface to form a three-dimensional gel system. Currently, hydrogel scaffolds are widely regarded as an ideal platform for stem cell culture and application, making them a prominent area of research. However, it is undeniable that hydrogel scaffolds may have limitations in terms of biocompatibility [18]. They show minimal effectiveness in promoting or prolonging the survival of cultured cells, and their degradation products may adversely affect cells, potentially shortening their lifespan. Zhou et al. proposed using natural human AM to create microcarriers called micronized AM (mAM) for expanding UC-MSCs in a 3D culture system [19]. Not only do AM have excellent biocompatibility to support cell proliferation, but they also provide a conducive microenvironment. mAM offers the advantage of a larger specific surface area compared to our approach, but both methods effectively extend and maintain cell lifespan and cell viability.

After 3 days of culture, FBs migrated directionally along collagen fibers (Figure 3c). Purified EpiSCs appeared small and round (Figure 3d), with a high nucleus-to-cytoplasm ratio. EpiSCs were uniformly inoculated on the surface of AM-FCG (Figure 3e), and after 7 days of culture, localized multilayering trends among different cells were observed (Figure 3f,g). H&E staining analysis revealed that the artificial skin exhibited a complete basal layer and a progressively differentiating granular layer structure, with a thin epidermal layer and densely arranged basal cells (Figure 3g). To explore the potential of AM-FCG-EpiSCs in promoting wound healing, *P63* immunofluorescence was further employed, as the *P63* protein plays a critical role in maintaining and regulating epidermal stem cells [20]. It was found that basal cells exhibited uniform expression of the stemness marker *P63* (Figure 3h), confirming that EpiSCs retained their stemness on the scaffold, mimicking the behavior and differentiation capacity of stem cells in natural skin. This suggests that the artificial skin possesses robust regenerative potential, which is essential for effective wound repair.

### 2.3. Morphological Characterization and Specific Protein Expression of the Biomimetic Skin

**Figure 4:**
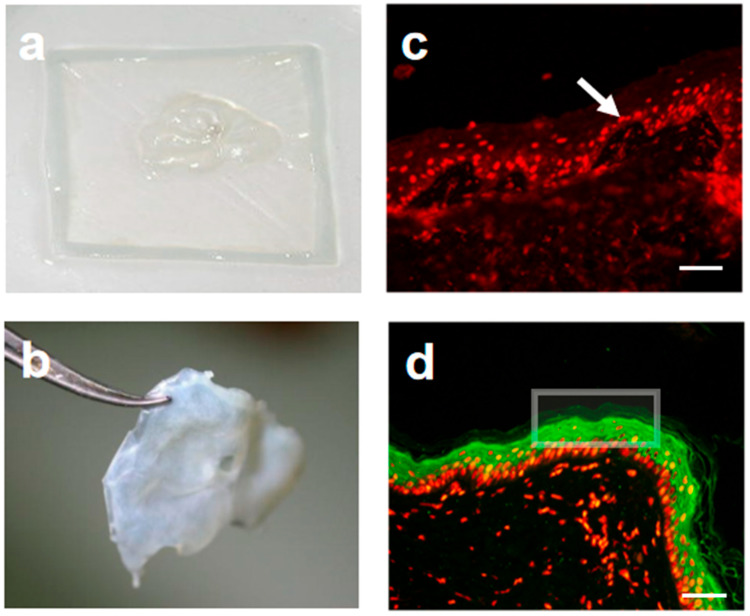
Morphological characterization and specific protein expression of the biomimetic skin: (**a**) initially cultured artificial skin; (**b**) fully cultured artificial skin; (**c**) Immunofluorescence analysis of initially cultured artificial skin. PI staining in red marks proliferative cells in the basal layer, with no positive CK1 signals in green; (**d**) Immunofluorescence analysis of artificial skin after prolonged culture. Green CK1-positive signals emerge in the suprabasal layers, confirming keratinocyte differentiation and epidermal stratification. The arrow indicates the nucleus stained with PI. The square marks the *CK-1* region. All scale bars represent 100 µm.

Similar to the spindle-shaped hydrogel devices created through 3D bioprinting [21], this study employs AM to constrain the longitudinal growth of organoids, providing the three-dimensional structure necessary for wound healing applications (Figure 3e). Furthermore, both experimental methods use components of the extracellular matrix, such as CG, to mimic the microenvironment of skin tissue, thus promoting cell growth and differentiation. Artificial skin was generated by spatially coordinating the differentiation of dermal and epidermal cells (Figure 4a). This was achieved through the physical separation of scaffold materials, FBs, and EpiSCs, while still allowing intercellular interactions. After in vitro maturation (Figure 4b), the artificial skin is ready for transplantation.

Immunofluorescence analysis was performed to evaluate cell proliferation and differentiation in the artificial skin. In initially cultured constructs, PI staining revealed abundant proliferative activity within the basal layer (red fluorescence, Figure 4c), while no obvious green fluorescence was observed, indicating the absence of terminal differentiation at this early stage. After prolonged culture, distinct CK1-positive signals (green fluorescence) appeared in the suprabasal layers, confirming the differentiation of keratinocytes and the formation of a stratified epidermis (Figure 4d). These results demonstrate that the artificial skin undergoes a sequential process of basal layer proliferation followed by terminal differentiation, closely mimicking the self-renewal and barrier functions of natural skin. Histologically, it successfully reconstructed the native skin architecture, with a stratified epidermis and underlying dermis. Further in vivo studies are needed to evaluate whether this engineered skin supports dermal matrix remodeling, angiogenesis, and innervation, which are key processes for full integration with host tissue. If confirmed, this strategy could offer a promising approach combining in vitro tissue engineering with in vivo integration for skin repair.

### 2.4. Scarless Wound Healing with Hair Follicles

**Figure 5:**
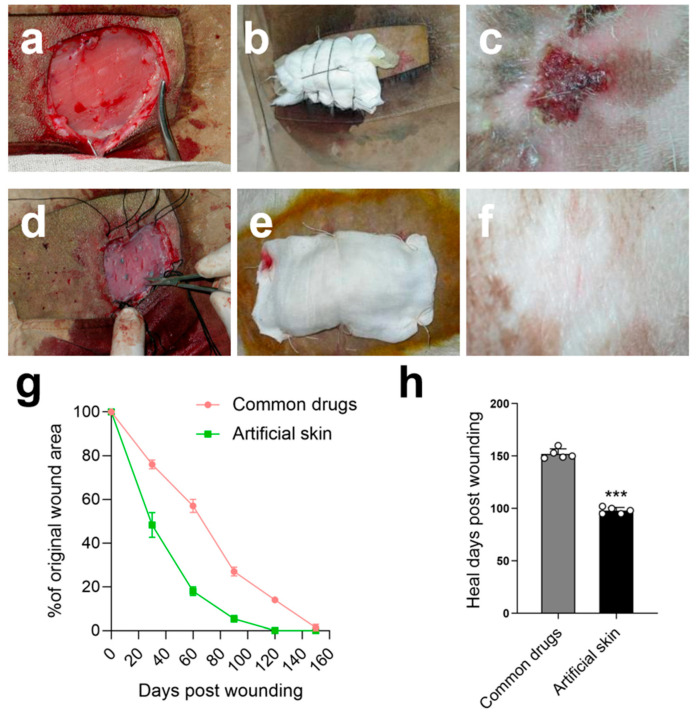
Recovery status of the wound section: (**a**) control group wound; (**b**) control group bandaging condition; (**c**) control group after sun exposure; (**d**) experimental group wound; (**e**) experimental group bandaging condition; (**f**) experimental group after sun exposure; (**g**) wound closure curves when treated with different groups; (**h**) average healed time of the wounds treated with different groups; Data presented as mean ± SD, *n* = 5, the error bars indicate the SD, statistically significant *** *p* < 0.001. Note: These images (**a**–**f**) are presented for qualitative morphological comparison only; no quantitative length analysis was performed.

Both the control and experimental groups underwent a 2.5 × 3 cm full-thickness skin excision procedure (Figure 5a). The control group received Moropicin ointment and was bandaged (Figure 5b), as the use of artificial skin AM-FCG-EpiSCs was designated as the experimental group (Figure 5d,e). In contrast to the porous bilayer artificial skin constructed by Xia et al., which emphasized a design of PLLA nanofibrous hydrophobic layer and carboxymethyl chitosan/gelatin lyophilized gel focusing on physical isolation and absorption functions [22], the AM-FCG-EpiSCs system developed in this study employs decellularized AM as a natural scaffold, emphasizing the simulation of the skin’s biological microenvironment and cellular self-organization capabilities. Although AM offers relatively limited physical protection, it functions effectively as a bioactive matrix wherein CG/FBs assemble into the dermal layer and EpiSCs self-organize to reconstruct the epidermal layer. The dynamic interaction between the natural extracellular matrix scaffold and cells helps address the issue of limited functional regeneration ability for chronic, full-thickness skin defects.

In our experiment, the experimental group of 2.5 × 3 cm wounds achieved a healing rate of 72.1% on day 60, while the control group had a healing rate of only 40.1%. By day 100, the wounds in the experimental group were almost completely closed, while the wounds in the control group remained open. After five months of treatment, the skin in both the experimental and control groups had healed. Although the wound areas of the control group had healed, we unexpectedly observed that, after subsequent continuous sun exposure of approximately 10 h of natural light daily for 30 consecutive days, the skin of the goats showed redness, peeling, and even bleeding after being exposed to the sun for a long time. However, we found no abnormalities in the experimental group (Figure 5f).

Although five months is long enough for Moropicin ointment to heal goat wounds, this result only represents a superficial outcome. The repaired skin in the control group failed to attain the protective effect of normal skin; no hair growth was observed, and it showed weak resistance to ultraviolet light. In contrast, the skin repaired in the experimental group was nearly as effective as normal skin, with the wound area well reconstructed and hair growth observed in certain regions. This study also indirectly proves the feasibility and high-quality effect of our artificial skin in wound repair. We are conducting further research on this.

**Figure 6:**
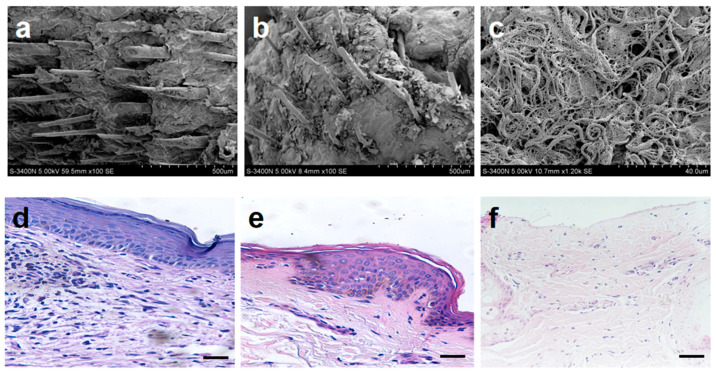
Analysis of micro-morphology and pathological structure in skin tissue repair: SEM images of the normal skin group (**a**), AM-FCG-EpiSCs treatment group (**b**) and Moropicin ointment treatment group (**c**); H&E images of the normal skin group (**d**), AM-FCG-EpiSCs treatment group (**e**) and Moropicin ointment treatment group (**f**). All scale bars represent 100 µm.

Recent studies have shown that FBs promote the thickening and elongation of epithelial cell layers and form hair follicle primordia through contractile forces. Additionally, they drive the remodeling of the basal layer by secreting substances, ultimately guiding the downward growth of hair follicle primordia to form fully developed hair follicles [23,24]. During wound healing, the cells at the edge of the wound need to be remodeled and differentiated into hair follicle cells. The mechanical forces, such as pressure and tension, generated during interactions between different cells may promote the proliferation and differentiation of hair follicle cells in this process. Observation of normal goat skin and skin wounds in the experimental group through SEM (Figure 6b, 100×) reveals that, although the hair on the skin wounds in the experimental group is less dense and mature compared to normal skin (Figure 6a, 100×), its morphology remains remarkably similar to that of normal hair.

In contrast, no obvious hair follicles were observed in the control group at this consistent magnification. At a higher magnification, the control group revealed a dense and disorganized network of collagen fiber bundles in the dermis (Figure 6c, 1200×), which was likely attributable to an unusually thin stratum corneum following Moropicin ointment treatment. Further verification by H&E staining revealed that the cells in the control group were loosely arranged and there were no obvious stratum corneum cells (Figure 6f). The cells in the experimental group were arranged more closely (Figure 6e). Injury-induced signals from the damaged tissue microenvironment activated the EpiSCs when we used AM-FCG-EpiSCs to treat the wound. These signals triggered the EpiSCs to proliferate and migrate to the wound site, where they primarily facilitated re-epithelialization, promoted wound closure, and ultimately restored skin integrity [25]. The experimental group was similar to normal skin (Figure 6d), presenting a distinct stratified structure, including the epidermis layer of the basal layer, spinous layer, granular layer and stratum corneum, as well as the dermis layer abundant in connective tissue.

### 2.5. Functional Restoration and Microstructural Remodeling

The synergistic effect of CG and AM provides a superior microenvironment for FBs and EpiSCs, facilitating faster and more complete tissue regeneration. CG has a guiding effect on FBs, EpiSCs and the aggregation of immune cells in the wound area, significantly accelerating the healing process [26,27]. Since the cells in the epidermal basal layer, known as EpiSCs, are the precursors of keratinocytes, the use of this artificial skin can indirectly promote the migration and proliferation of keratinocytes [28]. To better understand the potential of this artificial skin in promoting skin regeneration, we compared the expression levels of *CK1*, *CK10*, and *P63* in the repaired skin of both the experimental and control groups. Monitoring the changes in these molecules can provide a deeper understanding of the proliferation, differentiation, and migration of epithelial cells and the re-epithelialization of the wound bed during the wound healing process [29].

**Figure 7:**
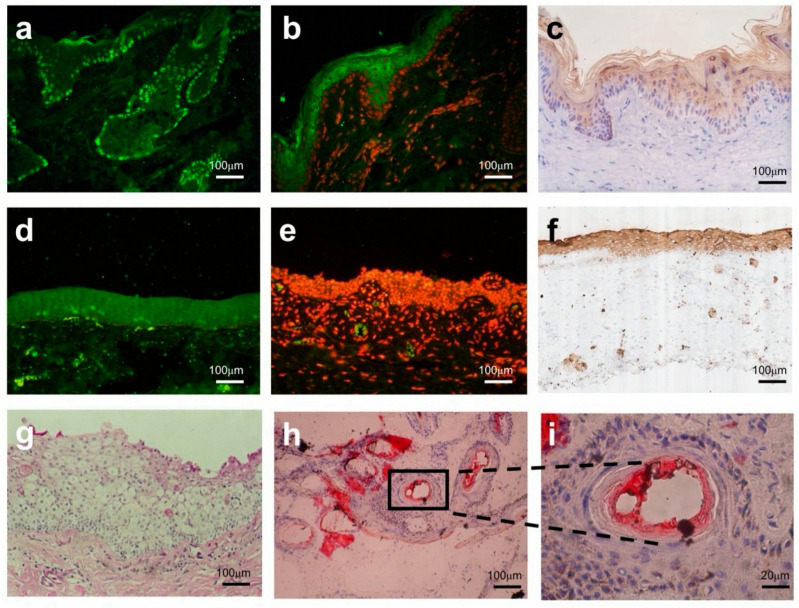
Histological analysis of key protein expression and lipid deposition in skin repair: (**a**) *P63* expression in the basal layer of the skin in the AM-FCG-EpiSCs treatment group; (**b**) *CK1* expression in the keratinized layer of the skin in the AM-FCG-EpiSCs treatment group; (**c**) *CK10* expression in the keratinized layer of the skin in the AM-FCG-EpiSCs treatment group; (**d**) absence of *P63* expression in the basal layer of the skin in the Moropicin ointment treatment group; (**e**) absence of *CK1* expression in the keratinized layer of the skin in the Moropicin ointment treatment group; (**f**) *CK10* expression in the keratinized layer of the skin in the Moropicin ointment treatment group; (**g**) Oil Red O staining of skin in the Moropicin ointment treatment group; (**h**) Oil Red O staining of skin in the AM-FCG-EpiSCs treatment group; (**i**) Enlarged view (5×) of the boxed region in (**h**). green in (**a**,**d**): P63; green in (**b**,**e**): *CK-1*; red in (**b**,**e**): nuclei.

The comparison results revealed that the basal layer of the regenerated epidermis in the experimental group presented dense *P63*^+^ cell nuclei (Figure 7a), confirming that the activity of EpiSCs remained effectively sustained after animal experiments with AM-FCG-EpiSCs. This suggests that this artificial skin is capable of maintaining a balance between proliferation and differentiation of basal cells in vivo. In contrast, the control group showed no specific *P63* signal (Figure 7d), indicating the depletion of its stem cell bank. The experimental group exhibited continuous and fragmented *CK1* signals in the stratum corneum (Figure 7b). Since *CK1* is an early marker of epidermal keratinocyte differentiation [30], this finding suggests that artificial skin can support the normal differentiation of epidermal cells and the formation of the stratum corneum. The *CK10* signal in the control group’s (Figure 7f) stratum corneum was sparse and discontinuous, contrasting sharply with the dense stratum corneum observed in the experimental group (Figure 7c). The high expression level of *CK1/10* in the experimental group indicates the recovery of the skin barrier function, which is crucial for preventing infection and dehydration.

Moropicin ointment primarily targets inflammation and immune responses. In the control group, only sporadic lipid droplets were observed in the dermis (Figure 7g), indicating potential suppression of adipocyte precursor activation and an absence of functional adipose tissue. This fat regeneration defect is consistent with the characteristics of scar tissue, which generally lacks a fat layer and shows elevated fibrotic ECM deposition [31]. Bioactive materials like the AM affect key processes such as lipid metabolism, immune regulation, and tissue remodeling by modulating paracrine signaling between EpiSCs and FBs [32,33]. A large number of lipid droplets were observed accumulating in the dermis of the experimental group (Figure 7h,i), and their structure was highly similar to that of physiological subcutaneous fat. Normal fat secretion is a key indicator of proper wound healing [34], which means that the damaged skin structure and function are returning to a state close to normal.

The growth factors of bone morphogenetic protein secreted by new hair follicles can convert FBs into adipocytes [35,36]. We speculate that AM-FCG-EpiSCs treatment successfully establishes a link between hair follicles and adipocytes, promoting adipocyte regeneration and normal secretion. In addition, the restoration of the fat layer helps reduce scar tissue formation, as adipose tissue serves a filling function in the later stages of wound healing, aiding in the recovery of both the appearance and function of the wound. The AM provides laminin to guide the morphogenesis of hair follicles. FBs and EpiSCs regulate paracrine signals through appropriate chemical signals to guide adipogenesis, remodel the appropriate microenvironment to induce sebaceous gland function recovery, and promote the regeneration of scar-free hair follicle tissue.

## 3. Conclusions

This study successfully developed a biomimetic composite skin system by integrating decellularized AM, fibroblast-enriched collagen gel, and EpiSCs, thereby constructing a novel engineered skin that combines both mechanical support and biological activity. This system not only mimics the multilayered structure of natural skin but also, through bidirectional intercellular regulatory mechanisms, coordinately promotes angiogenesis, inhibits scar formation, and supports epidermal regeneration and hair follicle development. Experimental results demonstrate that the biomimetic skin effectively promotes early closure and high-quality healing of full-thickness skin defects. Its histological structure, protein expression, and lipid metabolism closely resemble those of normal skin, and its performance is significantly superior to conventional treatment methods.

With its tunable component design and excellent biocompatibility, this system presents a promising strategy for advancing scarless repair and appendage regeneration. Furthermore, the integration of single-cell RNA sequencing and spatial transcriptomics could be employed to deeply investigate how the epidermis, dermis, and fat interact after AM-FCG-EpiSCs are implanted into wounds. This approach would provide novel theoretical foundations for the clinical translation and precise regulation of skin regeneration engineering.

## 4. Materials and Methods

Isolation and Identification of Cells: Tissue samples were harvested from the ear region (where blood vessels are relatively sparse) of donor Guanzhong dairy goats (female, 3 years old; bred at the Experimental Animal Farm of the Shaanxi National Stem Cell Engineering and Technology Research Center). The fur was shaved, and the area was disinfected prior to sampling. Using an ear punch, a skin sample approximately 0.5 × 0.5 cm^2^ in size was obtained and placed in physiological saline containing penicillin and streptomycin (1000 U/mL each). The sample was then transferred to a sterile environment. The skin was rinsed several times with PBS containing penicillin and streptomycin to remove any blood and hair. Under a dissecting microscope, the skin was separated from the cartilage and cut into smaller pieces measuring 0.2 × 0.2 cm^2^. We placed these pieces, epidermis-side up, in glass culture dishes measuring approximately 5.5 cm, using 3 to 4 pieces per dish. An appropriate amount of M199 culture medium supplemented with NBS, insulin, and other components as outlined in Table 1 was added. The dishes were then incubated in a carbon dioxide incubator (5% CO_2_, saturated humidity, 37 °C), with the culture medium being replaced every 2 days.

After 3–4 days, epithelioid cells had begun to grow from the edge of the tissue blocks. By 10 days, they had grown in a cobblestone-like pattern. On the layer of epithelioid cells, small round cells had aggregated, forming colony-like structures. When the diameter of the colonies reached 50 μm to 100 μm, they were picked under a dissecting microscope and treated appropriately with 0.25% trypsin and 0.02% EDTA. The cells were then dissociated and transferred using a glass pipette into 3.5 cm plastic culture dishes coated with gelatin, where they were cultured in serum-free medium. Cells derived from the primary culture and up to the 9th passage of stem cells will be subjected to cell slides. The study by Yang X et al. describes the detailed identification process of epidermal stem cells [37].

Preparation of FCG: Full-thickness skin samples were obtained from the same cohort of donor Guanzhong dairy goats (female, 3 years old; sourced from the Experimental Animal Farm of the Shaanxi National Stem Cell Engineering and Technology Research Center). The samples were disinfected, cleaned, and processed to remove the epidermis and subcutaneous fat to isolate dermal tissues. The dermal tissues were then cut into small pieces and subjected to enzymatic digestion to isolate FBs. The isolated cells were cultured in Dulbecco’s Modified Eagle Medium (DMEM; Lonza, Basel, Switzerland), supplemented with fetal bovine serum (FCS; Invitrogen, Carlsbad, CA, USA) and antibiotics (Lonza, Basel, Switzerland), as outlined in Table 1. Cultures were incubated at 37 °C with 5% carbon dioxide and 5% oxygen to promote cell proliferation and ECM secretion. We prepared FCG following the method for constructing human dermal equivalents (fdmDE) [38]. FBs were seeded into bovine-derived type I collagen solution at a specified density ranging from 2 × 10^5^ to 5 × 10^5^ cells/mL, and the resulting cell–collagen mixture, detailed in Table 2, was transferred to a gas-permeable culture dish for incubation. With cell proliferation and collagen deposition, the mixture gradually formed a stable gel-like structure, ultimately yielding an FCG.

AM Scaffold Preparation: Human amniotic membrane (HAM) was obtained from cesarean-derived placental sacs with negative serological tests. After an initial 6 h treatment in physiological saline containing triple antibiotics (0.1 U/mL penicillin, 0.1 U/mL streptomycin, and 0.1 U/mL gentamicin), the membrane was bluntly dissected under sterile conditions and thoroughly rinsed. Subsequently, 0.25% trypsin was added for cold digestion at 4 °C overnight to remove epithelial cells. The membrane was then placed in culture medium for 3 min, followed by repeated washing with PBS containing triple antibiotics. The processed AM was placed with its basement membrane facing upward in a glass dish, covered with a pre-fabricated hollow nitrocellulose membrane, and dried to obtain the AM scaffold. For long-term storage, the AM was immersed in pure glycerol and cryopreserved at −80 °C. Prior to use, it was rapidly thawed at 37 °C, washed with PBS containing triple antibiotics to remove glycerol, and then rehydrated in PBS under the same conditions in a 37 °C incubator for 45 min.

Biomimetic Skin Fabrication: The obtained fibroblast gel was evenly coated onto the AM scaffold and allowed to solidify for 2–3 days, resulting in the preparation of the amniotic membrane-fibroblast collagen gel composite scaffold (AM-FCG). The purified epidermal stem cells were then seeded onto the AM-FCG at a density of 3 × 10^6^ cells/cm^2^, and cell proliferation and differentiation were observed at appropriate time points. As shown in Table 1, the total duration of in vitro culture was 21 days, until a functional biomimetic skin was successfully constructed.

Immunofluorescence Staining: 0.5% Triton X-100 at room temperature for 10 min. Subsequently, blocking was performed with 5% bovine serum albumin (BSA, prepared in PBS) at 37 °C for 1 h. After removing the 5% BSA, the biomimetic skin was incubated with primary antibodies at 4 °C overnight. The primary antibodies used were anti-*P63* (EPR5701, Abcam, Cambridge, UK, diluted 1:200) and anti-*CK-1* (HPA017917, Sigma, Tokyo, Japan, diluted 1:300). The cells were then incubated with fluorescent secondary antibodies at room temperature in the dark for 1 h, followed by incubation with 0.5% DAPI solution for 5 min. Finally, an anti-fade mounting medium was applied to the cell droplet for coverslipping. The results were analyzed under a confocal microscope (Zeiss LSM 710, Oberkochen, Germany). All experimental samples were performed in triplicate. All immunofluorescence staining in this study followed the above procedure.

Skin Defect Model Animal: Full-thickness skin defect modeling was performed on recipient Guanzhong dairy goats (female, 3 years old; bred at the same facility as the donors). These recipient goats were siblings of the donor animals. They were fasted for one day prior to the transplantation surgery. During the surgery, 2.5 mL of the 846 mixture was administered via intramuscular anesthesia. The sheep was then secured in a lateral recumbent position on the surgical table. After symmetrically shaving and disinfecting the dorsal area, two full-thickness skin (including epidermis, dermis, and subcutaneous tissue) sections measuring 2.5 × 3 cm were surgically excised to create two acute skin wound sites labeled A and B. Gentle hemostasis was achieved using sterile gauze pressure.

The wound A (control group) was filled only with conventional anti-inflammatory ointments (Moropicin ointment), and 0.3 mg of the drug was applied to the skin per square centimeter once a day. The wound B (experimental group) was grafted with the previously prepared artificial skin patch. The patch was placed onto the wound bed and fixed with interrupted sutures at equal intervals, with appropriate edge overlap and tension control to ensure stable fixation. The surgical method was conducted in accordance with the method described in the literature by Bartholomew A [39]. Postoperatively, penicillin–streptomycin was administered intramuscularly daily for 3 days. The wound recovery was observed and recorded monthly. After 5 months, tissue samples from wounds A and B were taken for sectional observation.

Electron Microscopy: Scanning electron microscopy (Hitachi UHR FE-SEM SU8200, Tokyo, Japan) was used to observe the ultrastructure of skin samples from the normal skin group, the AM-FCG-EpiSCs treatment group, and the Moropicin ointment treatment group. Samples were subjected to dual fixation with 2.5% glutaraldehyde and 1% osmium tetroxide, dehydrated through a graded ethanol series, dried at the critical point, and sputter-coated with gold before being observed for surface morphology at an accelerating voltage of 5 kV. Five non-overlapping fields were randomly selected from each group to compare and analyze features such as hair follicle morphology and skin appendage structures. All operations were performed in triplicate, with imaging conditions kept consistent.

To further investigate the multilayering trends and ultrastructural features of the artificial skin construct, transmission electron microscopy was performed on samples harvested after 7 days of culture. Samples were fixed with 2.5% glutaraldehyde and 1% osmium tetroxide, dehydrated, and embedded in epoxy resin. Ultrathin sections (70–80 nm) were stained with uranyl acetate and lead citrate, and observed under a transmission electron microscope (TEM) at 80 kV. Images were captured to assess cell–cell contacts and epidermal stratification. At least five fields per group were examined to confirm multilayering.

Histological Assessment: All tissue samples were fixed in 4% paraformaldehyde, embedded in paraffin, and sectioned into consecutive 5 µm-thick slices for subsequent staining analysis. Each experimental group was performed in triplicate. The immunofluorescence staining procedures and analytical methods for *P63* and *CK-1* have been described in detail above. After dewaxing and rehydration, paraffin sections were subjected to H&E staining to assess skin tissue architecture. Nuclei were stained with hematoxylin for 5 min, differentiated with hydrochloric acid-ethanol, and cytoplasm, along with extracellular matrix, was stained with eosin for 1 min, followed by dehydration, clearing, and mounting with neutral gum. The stained sections were observed and photographed under a light microscope to evaluate epidermal integrity and dermal tissue structure.

Oil Red O Staining: Oil Red O staining was employed to assess skin lipid metabolism characteristics. Fresh tissue samples were embedded in optimal cutting temperature compound and cryosectioned into 8 µm-thick slices. After fixation, the sections were immersed in Oil Red O working solution (0.5% *w*/*v*) for 15 min, differentiated with 60% isopropanol, counterstained with hematoxylin, and mounted with glycerin gelatin. The stained sections were observed under a microscope, with positive lipid droplets appearing bright red. Five fields of view were randomly selected from each sample to observe the distribution of positive areas.

Statistical analysis: All experiments were performed at least in triplicate, and all experimental data were expressed as mean ± standard deviation (SD). Statistical analysis was performed using SPSS Statistics 31 (IBM, Armonk, New York, NY, USA). One-way analysis of variance (ANOVA) and two-tailed paired Student’s *t*-test were used to evaluate the difference in experimental data between groups. The *p* values ≤ 0.05 were considered statistically significant, and the *p* values ≤ 0.01 were considered highly statistically significant.

## Figures and Tables

**Table 1:** Culture medium formulations and culture durations.

Cell Type/Medium	Base Medium	Supplements	Concentration	Culture Durations
Fibroblast Medium	High-glucose DMEM with L-glutamine	Fetal Bovine Serum (FBS)	10% FBS	Fibroblasts: 5 days
		Penicillin-Streptomycin	1% Pen-Strep (100 U/mL)	
EpiSC Medium	M199	Newborn Calf Serum (NBS)	15–20% NBS	EpiSCs (primary isolation): 10 days
		Insulin	5–8 μg/mL	
		Hydrocortisone	0.5 μg/mL	
		Penicillin-Streptomycin	100 U/mL	
AM-FCG-EpiSC Medium	DMEM/F12	Ascorbic Acid (Vitamin C)	40–50 μg/mL	AM-FCG-EpiSCs (co-culture for biomimetic skin construction): 21 days
		DMEM with 20% FBS	10% (*v*/*v*)	
		Insulin	5 μg/mL	
		EGF	20 ng/mL	
		IGF-1	20 ng/mL	

**Table 2:** Preparation of AM-FCG.

Component/Step	Volume Ratio	Original Concentration	Final Concentration/Content
Collagen Solution	7 volumes	4.5 mg/mL	3.15 mg/mL
5× DMEM Medium	2 volumes	5× concentrated solution	1× working solution
Conditioned Medium (with cells)	1 volume	5 × 10^6^ cells/mL	5 × 10^5^ cells/mL (in gel)
1M NaOH	as needed	-	adjust pH to 7.2

## Data Availability

The data presented in this study are available upon request from the corresponding author. The data are not publicly available due to ethical reasons.
